# Quantitative characterization of the long-term charge storage of a ZnO-based nanorod array film through persistent photoconductance†

**DOI:** 10.1039/c8ra02318b

**Published:** 2018-05-04

**Authors:** Linzhi Lu, Xiaotong Jiang, Huiqiong Peng, Dawen Zeng, Changsheng Xie

**Affiliations:** State Key Laboratory of Material Processing and Die & Mould Technology, Nanomaterials and Smart Sensors Research Laboratory, Department of Materials Science and Engineering, Huazhong University of Science and Technology Wuhan 430074 PR China csxie@mail.hust.edu.cn +86-27-8754-3778 +86-27-8755-6544

## Abstract

The persistent nature of the increased conductivity upon removal of incident illumination, described by the term persistent photoconductivity (PPC), in ZnO films is sensitive to their defect states. PPC can be viewed as a process of charge storage with relevant defects. To evaluate charge storage quantitatively, in this work, some thought-provoking characteristic quantities were derived from a photocurrent–time curve acquired by testing the photoelectric properties of ZnO under on and off UV illumination. *Q*_uo_ was defined as the obtained charge number per unit voltage during the light-on phase, while *Q*_us_ was defined as the storage charge number during the light-off phase. *η* was acquired by dividing *Q*_us_ by *Q*_uo_ to measure the storage efficiency after the removal of UV light. On the basis of previous work, it was assumed that the PPC of ZnO originated from the unique property of V^0^_O_. Meanwhile, this report reveals that the intrinsic defects V_O_^2+^, V_O_^+^, V^0^_Zn_ will enhance *Q*_uo_ and *Q*_us_ but decrease *η* in the pure ZnO nanorod array film. The extrinsic defect Cu^0^_Zn_ introduced by coating the ZnO nanorod array film in an ethanol solution of copper acetate suppresses *Q*_uo_ and *Q*_us_ but promotes the increase of *η*. Since the whole methodology originated from a series of physical definitions, it can be easily extended to other materials with similar PPC effects.

## Abstract 2

*Q*_uo_, *Q*_us_ and *η* were derived from a photocurrent–time curve to quantitatively characterize the charge storage of a ZnO-based nanorod array film.

## Graphical abstract

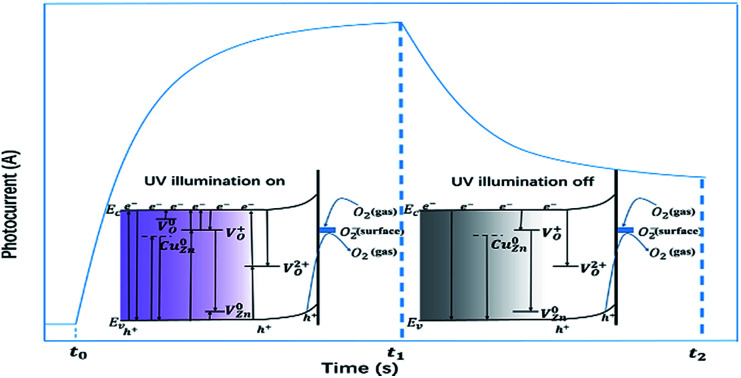

## 1 Introduction

The unique phenomenon of the long relaxation of photoconductance after removing illumination observed in many semiconductor crystals (GaN,^1,2^ SiC,^3^ ZnO,^4,5^ SnO_2_,^6^ Bi_2_S_3_,^7^ WO_3_,^8^), known by the term persistent photoconductivity (PPC), has stimulated the profound interest of researchers. Many investigations have been carried out to clarify the origin of the phenomenon of persistent photoconductivity, with the following hypotheses being predominant. For one thing, some authors claim that the existence of defects which exhibit a metastable charge state is responsible for this property.^9–12^ The reverting of excited electrons to their ground state is impeded by a thermally activated barrier generated from structural relaxation when the illumination is switched off, thus resulting in PPC. Alternatively, theorists who disagree with the former assumption invoke a built-in electric field engendered by the surface properties of metal oxides to explain the observed phenomenon.^4,8,13^ The ability to separate electrons and holes at the negative electric field facilitates the transport of holes to the surface, lengthening the lifetime of the electrons and giving rise to PPC.

In spite of the disagreement, both theoretical models agree that PPC inherently involves the recombination and storage of photogenerated carriers which are extremely sensitive to microstructure, including the morphology and defects or impurities. In Lee’s work, nanoporous GaN formed by electrochemical etching demonstrated enhanced persistent photoconductivity for the stronger built-in electrical field due to more absorbed oxygen.^14^ In Zhu’s work, no PPC appeared in ZnO nanoparticles but it did appear in ZnO nanorods, which was attributed to the different carrier transport mechanisms resulting from their different morphologies.^15^ A WO_3_ film with a needle-like structure exhibited significantly improved persistent photocurrent compared to a WO_3_ nanocrystalline film.^16^ Also, the effect of defects on the PPC could be complicated depending on the position of the energy levels of defects. Generally, defects with energy levels close to the midgap tend to assist the recombination of carriers. Meanwhile, those with an energy level approaching the Fermi level are supposed to trap a minority of the carriers, prolonging the lifetime of the majority of the carriers. Evidently, the former will weaken the PPC whereas the latter enhances it. Villafuerte reported zinc vacancies compensating donors acted as trapping centres and the V_Zn_–V_O_ divacancies of ZnO in the surface of the microwires acted as recombination centres that steeply reduced the photoconductivity.^17^ Prades *et al.* observed that the PPC of individual metal oxide NWs was rapidly reverted after increasing the oxygen content in air, revealing the recombination through surface defects favoured by oxygen.^18^

As mentioned above, previous work concerning PPC focuses mostly on either figuring out a model to explain the phenomenon or blocking the PPC, which dramatically damages the performance of optoelectronic detectors and gas sensors. As a matter of fact, the persistent nature of the increased conductivity implies that part of the photogenerated carriers still remains in an excited metastable state, or in other words, is stored in the illuminated semiconductor upon removal of the incident illumination. However, few reports concentrate on the nature of PPC as a process involving the long-term storage of carriers, which can be further understood as the storage of optical energy, whereas in this report new insight into the storage role of defects over the PPC will be provided. The photogenerated carriers are carefully probed to develop the potential of energy storage for those materials possessing PPC. The most important challenge is how to establish a method for the quantitative characterization of long-term carrier storage. Therefore, a method for the quantitative characterization of long-term carrier (holes for p-type semiconductors and electrons for n-type semiconductors) storage through persistent photoconductance is proposed for the first time based on the physical definitions of current. To characterize the quantity of carriers, the integral area of a photocurrent–time curve both in photoresponse and decay periods is calculated in detail. Furthermore, a series of essential values are also defined to measure the capacity of charge storage. The effect of the defects on these essential values (*Q*_uo_, *Q*_us_ and *η*) is discussed. Here, *Q*_uo_ was defined as the obtained charge number per unit voltage during the light-on phase while *Q*_us_ was defined as the storage charge number during the light-off phase. *η* was acquired by dividing *Q*_us_ by *Q*_uo_ to measure the storage efficiency after the removal of UV light. To confirm the validity of this method, pure ZnO and copper acetate coated ZnO nanorod array films were successfully synthesized through a wet chemical method. Due of the existence of various intrinsic defects in the former and extrinsic defects in the latter, we can comprehensively investigate the role of the defects in long-term carrier storage. Undoubtedly, with increasing environmental degradation and the rapid consumption of natural resources, exploiting the relationship between the PPC and charge storage of optical energy is of significant importance in various fields, such as realizing more effective solar power generation, the degradation of hazardous substances and the conversion of solar energy into fuels in the dark.

## 2 Experimental

### 2.1 Method for the quantitative characterization of long-term charge storage

**Fig. 1:**
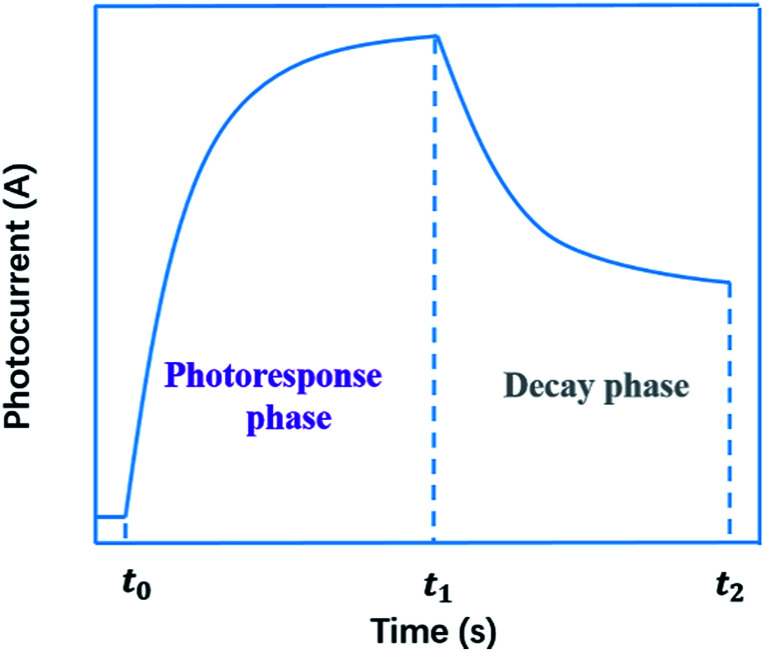
A typical curve of photocurrent *vs.* time.

Generally, the photoconductivity was determined from the total current under illumination after subtracting the dark current. The samples were excited using an optical pulse generated by various kinds of light source with emissions ranging from ultraviolet to near-infrared. In the obtained curve of photoconductivity *vs.* time, or photocurrent *vs.* time, there are two phases, *i.e.*, the photoresponse in the stage of illumination-on and the decay in the stage of illumination-off. In the photoresponse phase, the photoconductivity, or photocurrent, first increases quickly, then decreases its rising speed until it approaches a saturation level. In the decay phase, the conductivity, or current, still remains at a high level and decreases slowly. A typical curve of photoconductivity *vs.* time, or photocurrent *vs.* time, is shown in Fig. 1. As shown in the decay phase, the persistent nature of the increased conductivity upon removal of the incident illumination is described by the term persistent photoconductivity (PPC).

To obtain the characteristic values for the quantitative characterization of long-term carrier storage, we focused on mathematical treatment of the photocurrent–time curve based on general physics. As is well known, current is defined as the quantity of electric charge across a conductor section in a unit time, *i.e.*

1 *I* = d*q*/d*t*

where *q* is the number of charges passing through the cross section of the conductor during the time period of *t*.

According to eqn (1), the number of charges for the photoresponse phase (*Q*_o_) can be obtained by integrating the *I*–*t* curve in the time interval of the stage of illumination-on,2
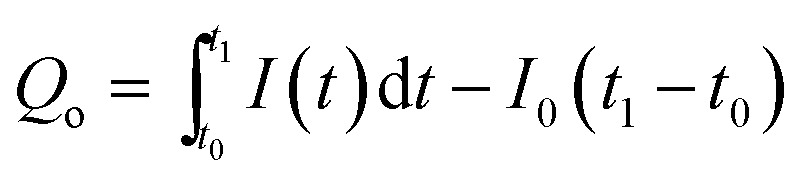
while the number of charges for the decay phase (*Q*_s_) can also be given by integrating the *I*–*t* curve in the time interval of the stage of illumination-off as follows,3
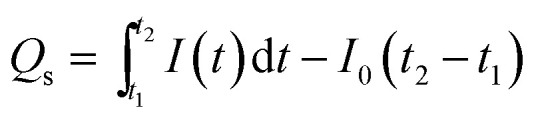
here, the number of charges for the photoresponse phase (*Q*_o_) is the total number of photogenerated carriers, while the number of charges for the decay phase (*Q*_s_) might be considered as the number of stored charges in a sample after illumination-off.

Since the photocurrent measurement was performed under different bias voltage, the number of charges per unit voltage (*Q*_uo_) in the photoresponse phase and the number of stored charge per unit voltage (*Q*_us_) in the decay phase are employed to eliminate the impact of voltage (*U*).

4 *Q*_uo_ = *Q*_o_/*U*

5 *Q*_us_ = *Q*_s_/*U*

Thus, an important parameter to characterize the storage efficiency (*η*) is established by following expression,

6 *η* = *Q*_us_/*Q*_uo_

Obviously, the total number of photogenerated carriers (*Q*_uo_), the number of stored charges (*Q*_us_) and the storage efficiency (*η*) are novel feature parameters which can quantitatively characterize the long-term charge storage through persistent photoconductance. Using these novel feature parameters, we can further understand the role of various kinds of defects in long-term carrier storage and persistent photoconductance.

### 2.2 Preparation of the ZnO nanorod array films

The process for cleaning the ablated fluorine doped tin oxide (FTO) glass (from Nippon Sheet Glass Company in Japan) and the synthesis of the ZnO nanorod array films followed the methods described in ref. 15. 0.1 mol of zinc acetate Zn(CH_3_COO)_2_·2H_2_O was dissolved in 200 mL of absolute methanol. The obtained solution was stirred with a magnetic stirrer for 1 h, then 6 mL of monoethanolamine C_2_H_7_NO was added into the solution. The solution was aged for 24 h at room temperature after stirring for 90 min. A dip coating method was used to coat the sol on the FTO electrodes which had been cleaned and dried. The dip coater (ZR-4200) was made by Zhongrui Instrument Co, LTD, China. The drawing rate and dipping rate were 100 mm min^−1^ and 200 mm min^−1^ respectively. Every sample was coated twice in order to get sufficient ZnO seed layers. The samples were dried at 90 °C in air for 30 min after each coating. Finally, the obtained films were calcined in air at temperatures up to 500 °C.

The heating rate was 3 °C min^−1^, and the holding time was 1 h. Then the ZnO nanorods were grown on the nanoparticle seed layers by an aqueous solution method. The growth solution was made by mixing 0.02 mol Zn(CH_3_COO)_2_·2H_2_O and equi-molar C_6_H_12_N_4_ precursors in 1 L deionized water. A mixed precursor was obtained after magnetic stirring for 1 h. The seed-coated FTO electrodes were put into a closed jar which contained the growth solution. The jar was kept in a 90 °C thermostat water bath for 4 h. Then the samples were taken out and rinsed with deionized water several times in order to remove any possible vestigial ions. Finally, the ZnO NRs samples were dried at 80 °C in air for a day.

All chemicals purchased from Guoyao Chemical Reagent Co. LTD were of analytical reagent grade and used without further purification.

### 2.3 The synthesis of the Cu(CH_3_COO)_2_-coated ZnO nanorod array films

2 mmol Cu(CH_3_COO)_2_·2H_2_O was dissolved in 100 mL absolute ethyl alcohol. After being stirred with magnetic stirring for 1 h, a blue homogeneous solution was obtained. A dip coating method was used to coat the solution on the FTO electrodes on which the ZnO nanorods had grown. Every sample was coated twice and dried at 80 °C in air for 30 min after each coating. The drawing rate and dipping rate were 100 mm min^−1^ and 200 mm min^−1^ respectively. Finally, the obtained samples were calcined in air at various temperatures (400 °C, 500 °C and 600 °C) for 90 min. The heating rate was 3 °C min^−1^. Pure ZnO nanorod array films prepared with the same heat treatment were also produced and labelled as S400-1 (annealed at 400 °C), S500-1 (annealed at 500 °C), S600-1 (annealed at 600 °C). S400-2, S500-2 and S600-2 were used to mark the Cu(CH_3_COO)_2_-coated ZnO nanorod array films annealed at 400 °C, 500 °C and 600 °C, respectively.

### 2.4 Structural characterization and photocurrent measurement

An X-ray diffractometer (X′ Pert PRO, PANalytical B.V.) using Cu K_α1_ radiation was used to identify the phase composition of the samples. The morphology of each sample was observed by field emission scanning electron microscopy (Hitachi S-4800 FESEM). The PL spectra of the samples were acquired on a laser confocal microscope Raman spectrometer (LabRAM HR800) with a 30 mW He–Cd laser (325 nm) at room temperature. The surface chemical analysis of the composition was studied by X-ray photoelectron spectroscopy (XPS, VG Multilab 2000). All obtained XPS spectra were calibrated to a C 1s electron peak at 284.6 eV.

Photoconductive testing was performed under a dry air flow at room temperature on a test platform designed by our laboratory. Given the minimum current that the test platform could probe accurately, the samples were set at a proper bias (shown in Table 1) at 15 s in order to get the electric signals and then exposed to an ultraviolet (UV) LED array (Light Emitting Diode, Shenzhen Ti-Times Co.) at 30 s for 300 s, which was called the photoresponse phase. Then the UV illumination was turned off for 240 s, which was termed as the decay phase. Curves of photocurrent *vs.* time for the tested samples were obtained.

**Table 1:** Testing bias voltage of all the samples

Testing bias voltage of all the samples (V)					
S400-1	S500-1	S600-1	S400-2	S500-2	S600-2
0.01	0.01	0.01	5	5	0.5

## 3 Results and discussions

### 3.1 Structure and morphology

**Fig. 2:**
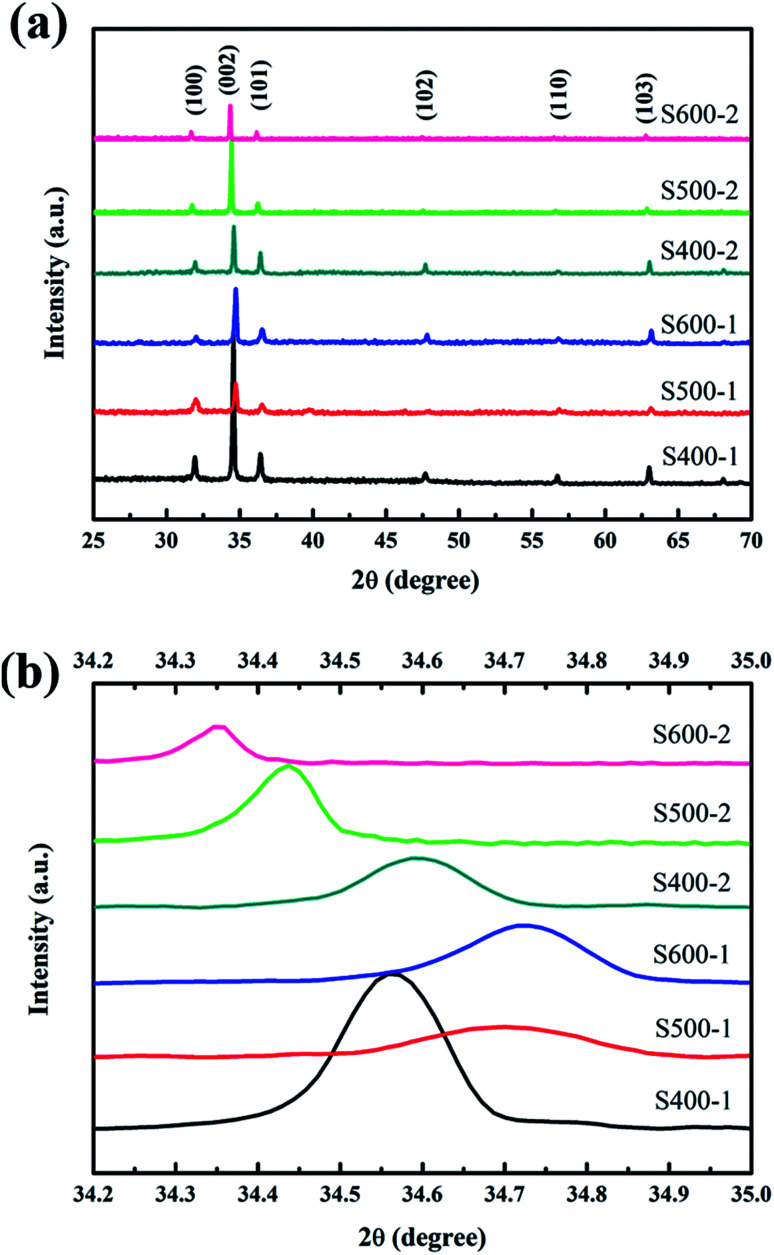
(a) XRD patterns of all the samples; (b) the diffraction angles of the (0 0 2) plane for all the samples.

Fig. 2(a) shows the XRD patterns of the ZnO nanorod array films annealed at different temperatures (400 °C, 500 °C and 600 °C). All the peaks are indexed from the hexagonal wurtzite zinc oxide (JCPDS 36-1451), while no peaks of CuO or impurities were detected. The distinguishable diffraction peaks correspond well to the (1 0 0), (0 0 2), (1 0 1), (1 0 2), (1 1 0) and (1 0 3) crystal planes of hexagonal ZnO. An intense (0 0 2) preferred orientation is observed for all the samples, which implies the 〈0 0 2〉 orientation growth was enhanced. Fig. 2(b) shows the variation in peak position along the (0 0 2) plane. With increasing annealing temperature, the peak position of the (0 0 2) plane in pure ZnO nanorods shifts towards higher angles, which is linked to the formation of more vacancies resulting in the decrease of the lattice constant. The slightly higher angle of the Cu(CH_3_COO)_2_-coated ZnO nanorods annealed at 400 °C compared with the pure ZnO nanorods indicates the substitution of the Zn^2+^ ions by smaller Cu^2+^ ions. The further decrease in the diffraction angles of the Cu(CH_3_COO)_2_-coated ZnO nanorods might be associated with the entry of Cu atoms into the Zn vacancy sites.^19^

**Fig. 3:**
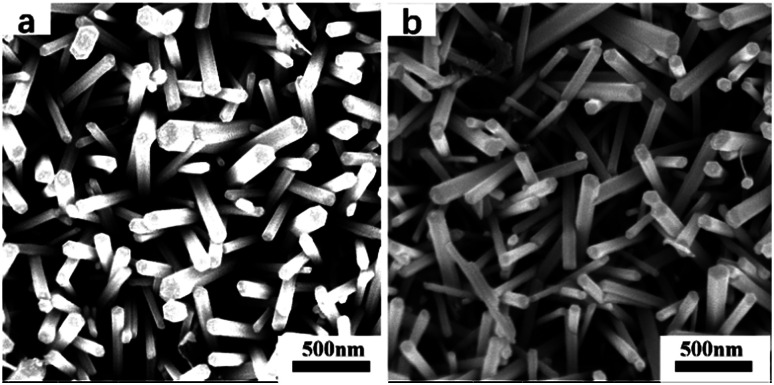
FESEM images of the samples annealed at 400 °C: (a) the pure ZnO nanorod array film; (b) the Cu(CH_3_COO)_2_-coated ZnO nanorod array film.

The morphologies of the samples annealed at 400 °C are presented in Fig. 3. As shown in Fig. 3a and b, both samples acquired rod-like morphologies from the aqueous solution method, with an average diameter of 100 nm and a length of ∼1 μm. There was no obvious difference in morphology between the Cu(CH_3_COO)_2_-coated ZnO nanorods and the pure ZnO nanorods, indicating that the addition of copper acetate had no distinguishable influence on the morphology of the ZnO nanorods. FESEM was also used to discover the morphologies of the samples annealed at 500 °C and 600 °C. It turned out that no obvious changes took place. Thus the results are not displayed here.

### 3.2 XPS characterization

**Fig. 4:**
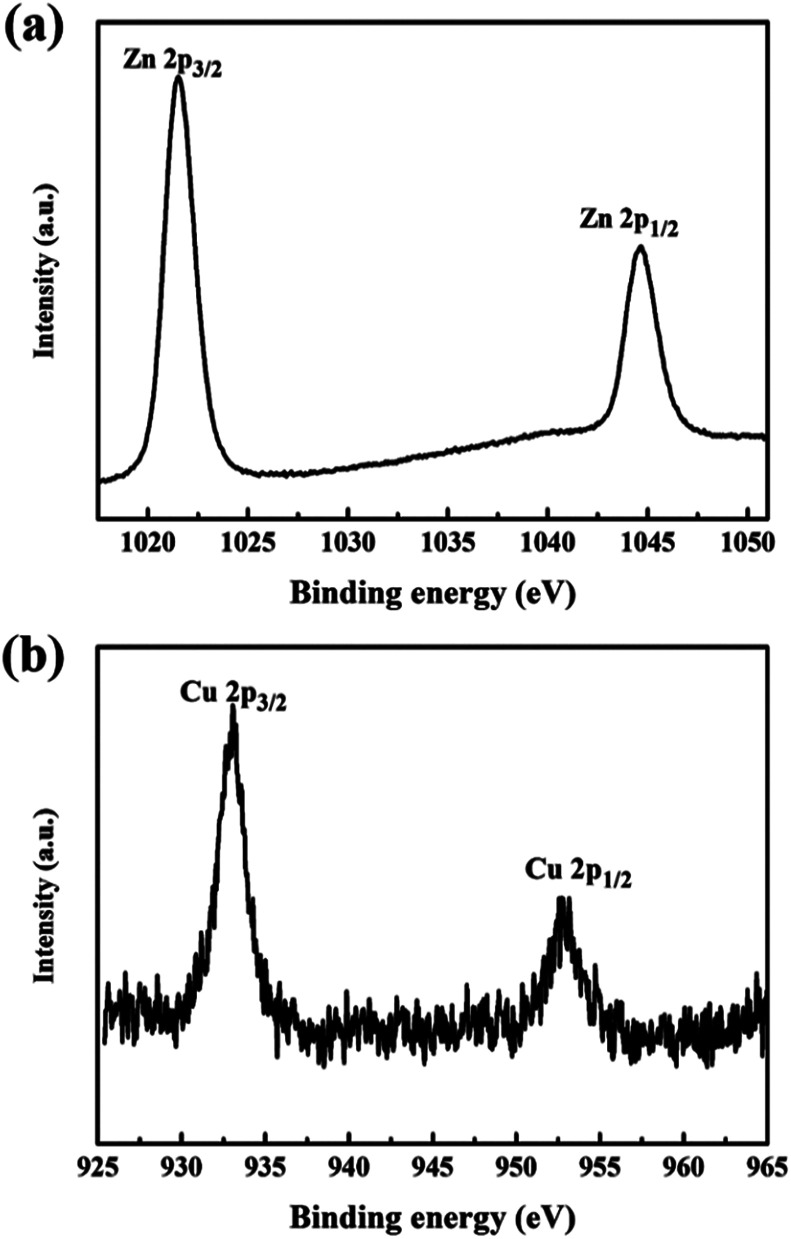
High resolved XPS survey spectrum of (a) Zn-2p in the pure ZnO nanorods and (b) Cu-2p in the Cu(CH_3_COO)_2_-coated ZnO nanorods (both samples were annealed at 400 °C).

Chemical state and surface information was collected by XPS. Fig. 4 shows the high resolved XPS survey spectrum of (a) Zn-2p in the pure ZnO nanorod array film and (b) Cu-2p in the Cu(CH_3_COO)_2_-coated ZnO nanorod array film (both samples were annealed at 400 °C). The peaks at 932.92 eV and 952.79 eV belong to Cu-2p_3/2_ and 2p_1/2_ respectively.^20,21^ The shakeup satellite peaks typical of Cu^2+^ are too weak to be observed. All the Cu(CH_3_COO)_2_-coated ZnO nanorods samples present a similar XPS spectrum of Cu-2p, unambiguously confirming the presence of Cu^2+^. The peaks at 1044.66 eV and 1021.57 eV corresponding to the Zn-2p_3/2_ and 2p_1/2_ core levels indicate the presence of Zn^2+^.^22–24^

An attempt to separate the O 1s spectra into several subspectral components was made (shown in the ESI†), centred at 529.97 ± 0.04 eV (Oa), 530.4 ± 0.3 eV (Ob), 531.37 ± 0.3 eV (Oc), and 532.30 ± 0.25 eV (Oe), respectively. The low binding energy components located at Oa and Ob are attributed to the O^2−^ ions on the lattices surrounded by copper atoms and zinc atoms with a full supplement of nearest-neighbour O^2−^ ions separately; Oc is assigned to the O^2−^ ions in the oxygen deficient regions of ZnO and CuO; the Oe peak of the O 1s spectrum is related to the absorption of a specific species, such as adsorbed O_2_ or adsorbed H_2_O, –CO_3_.^25,26^

**Fig. 5:**
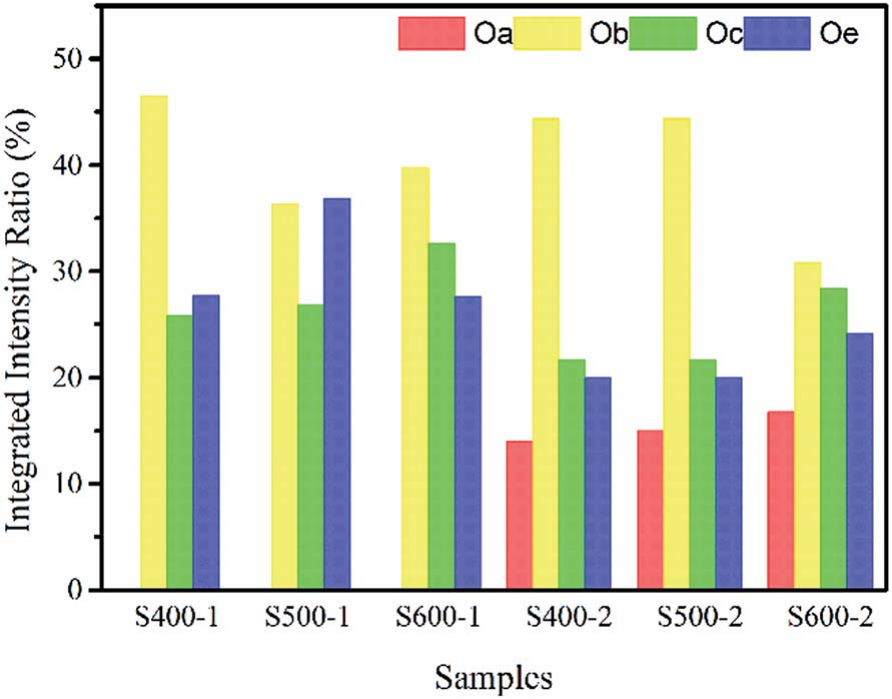
Integrated intensity ratio of different peaks in each sample.

The integrated intensity ratios of each peak are given in Table 2 and Fig. 5. As we can see, the integrated intensity ratio of Oc both in the pure ZnO nanorods and the Cu(CH_3_COO)_2_-coated ZnO nanorods increases when the annealing temperature rises, indicating more O-related defects come into being because of the immigration of the O atoms to the surface. Also, the increase in the integrated intensity ratio of Oa indicates that more Cu atoms entered the Zn lattices and more CuO was likely to be produced on the surface of the ZnO nanorods by the annealing of the coated Cu(CH_3_COO)_2_. Obviously, the changes in the integrated intensity ratio of Oc verify that the Cu(CH_3_COO)_2_ coating altered the surface state.

**Table 2:** Integrated intensity ratio of different peaks in each sample

Banding energy	Integrated intensity ratio of different peaks in each sample (%)					
	S400-1	S500-1	S600-1	S400-2	S500-2	S600-2
Oa	—	—	—	14.03	14.99	16.77
Ob	46.51	36.35	39.73	44.33	37.11	30.80
Oc	25.83	26.78	32.65	21.65	26.47	28.34
Oe	27.66	36.87	27.61	19.99	21.43	24.09

### 3.3 Photoluminescence properties

**Fig. 6:**
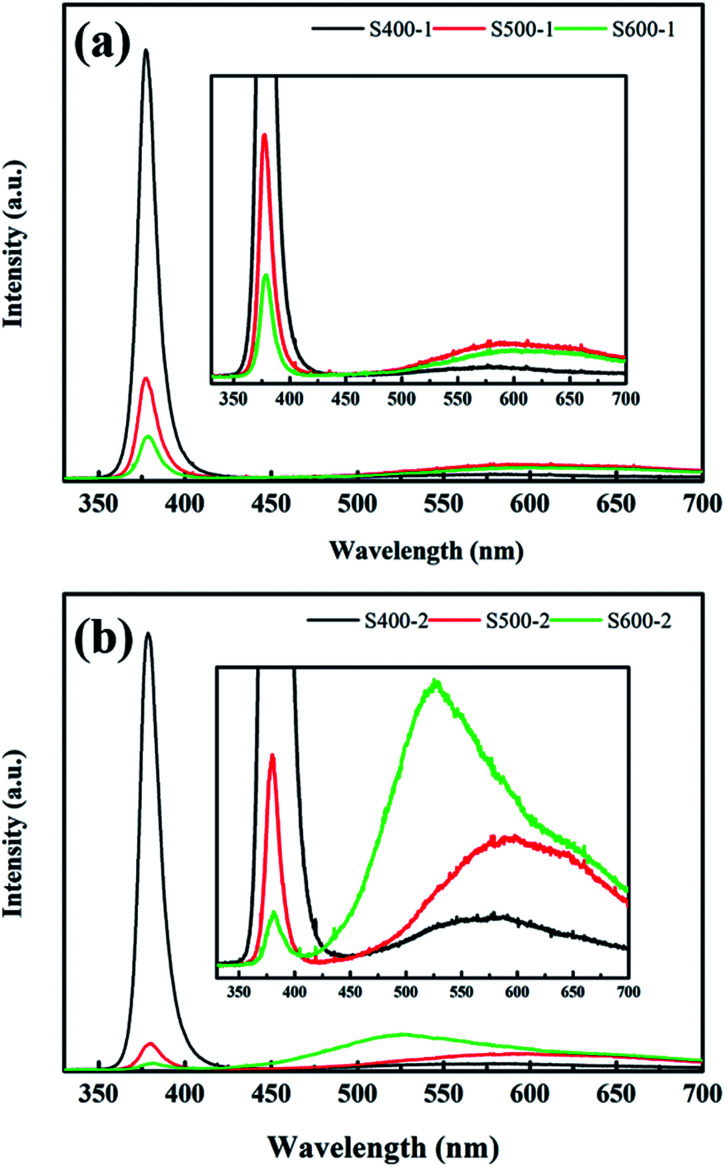
PL spectra of (a) the ZnO nanorods; (b) the Cu(CH_3_COO)_2_-coated ZnO nanorods annealed at different temperatures.

PL measurements are a powerful tool to detect emission properties to clarify the related defect state of semiconductor metal oxides. Fig. 6 shows the room temperature PL spectra of the pure ZnO nanorods and the Cu(CH_3_COO)_2_-coated ZnO nanorods recorded in the range 350–700 nm with a 325 nm HeCd laser as a pump source. In general, all spectra exhibit a typical UV emission peak and a broad visible light emission extending from about 500 nm to 700 nm. The UV emission centred at 380 nm is attributed to a near-band-edge (NBE) transition of ZnO,^27^ while various defects account for the visible light emission. It is common that the pure ZnO nanorods and the Cu(CH_3_COO)_2_-coated ZnO nanorods annealed at 400 °C display extraordinarily strong UV emission with negligible visible light emission. However, as the annealing temperature increases, the intensity of the UV emission decreases sharply while the visible light emission is obviously enhanced, which is potentially due to the higher density of defects since increasing the temperature is able to provide enough energy for the formation of defects. It is also noteworthy that coating ZnO in Cu(CH_3_COO)_2_ largely increases the visible light emission, which even surpasses the UV emission for the Cu(CH_3_COO)_2_-coated ZnO nanorods annealed at 600 °C. Undoubtedly, new defects appear in the composite but remain to be explored.

In order to further understand the impact of the annealing temperature and the Cu(CH_3_COO)_2_ coating on the defects, the PL spectra of the pure ZnO nanorods were resolved into three different peaks (shown in the ESI†) through Gaussian fitting: UV emission (380 nm), green-yellow emission (567–600 nm), and red emission (652–669 nm). Not surprisingly, an emerging green emission (520–538 nm) exists for the Cu(CH_3_COO)_2_-coated ZnO nanorods, which is likely linked to the doping of Cu. The intrinsic defects of ZnO are well documented in previous reports, including V_O_, Zn_i_, Zn_O_, V_Zn_, O_i_, and O_Zn_. Among the first three donor defects, V_O_ is the most prevalent with the lowest formation energy. The acceptor defect with the lowest formation energy is V_Zn_. Oxygen vacancies can occur in three different charge states: the doubly ionized oxygen vacancy V_O_^2+^, the singly ionized oxygen vacancy V_O_^+^, and the neutral oxygen vacancy V^0^_O_. According to previous research, the energy levels of V_O_^2+^ and V_O_^+^ are located at 1.6 eV^11^ and 2.34–2.53 eV (ref. 28) above the VBM respectively, making them the deep defects that assist the recombination of carriers as well as the effective light emission centres. In contrast, V^0^_O_, whose energy level is close to the conduction band, serves as the electron trap and contributes nothing to the light emission. V_Zn_ is an acceptor with a transition level *ε*(0/−) = 0.18 eV.^29^ In such a scenario, the red emission is caused by the recombination of electrons from the CB and the capturing of holes by V_O_^2+^ and the yellow emission results from the donor–acceptor transition involving V_O_^+^ and V_Zn_. In other cases where green emission is observed in pure ZnO, the transitions related to the single ionized oxygen vacancies,^28,30,31^ the zinc interstitials,^32^ and the Zn vacancies^33^ are responsible for the appearance of green light. While in our work, green emission accounts for the doping of Cu in ZnO, discovered by exhaustively comparing the results of PL and XPS with the pure ZnO nanorods samples. Besides, much outstanding research has explicitly stated that copper implantation in ZnO gives rise to green emission.^34–36^

The relative integrated intensity ratios of all the emission peaks were also calculated and listed in Table 3. As shown, the ratio of UV emission for both the pure ZnO nanorods and the Cu(CH_3_COO)_2_-coated ZnO nanorods decreases sharply with increased annealing temperature which favours the formation of defects. Meanwhile, the higher annealing temperature promotes more Cu to diffuse into the ZnO to substitute for Zn, causing stronger green emission. The diminution of the yellow emission in the Cu(CH_3_COO)_2_-coated ZnO nanorods corresponds to the decrease of V_Zn_ due to the entrance of Cu into V_Zn_.

**Table 3:** Relative integrated intensity ratio of different emission peaks in each sample

Emission peak	Relative integrated intensity ratio of different emission peaks (%)					
	S400-1	S500-1	S600-1	S400-2	S500-2	S600-2
UV	90.48	35.32	23.60	86.09	12.82	2.24
Green	—	—	—	6.09	19.92	57.68
Yellow	7.15	41.32	40.31	3.59	29.02	20.34
Red	2.37	23.36	36.09	4.23	38.24	19.74

### 3.4 Photoelectric properties

**Fig. 7:**
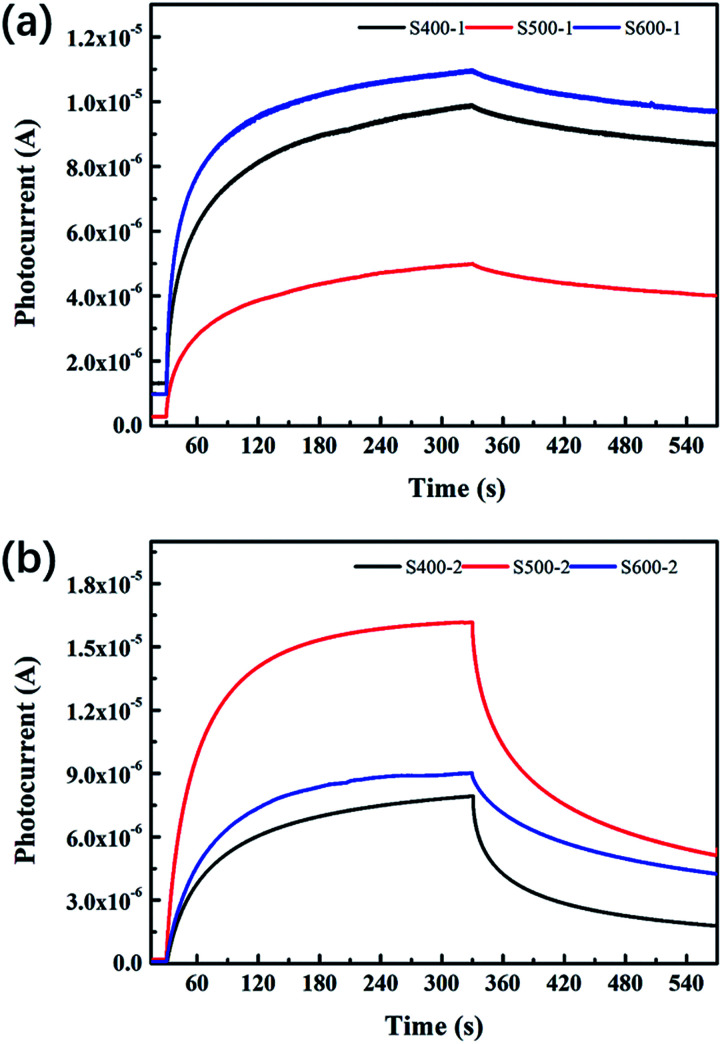
Photocurrent–time curves of (a) the pure ZnO nanorods (b) the Cu(CH_3_COO)_2_-coated ZnO nanorods.

The photocurrent–time curves for all the samples are exhibited in Fig. 7. Table 4 clearly presents detailed information about the *Q*_uo_, *Q*_us_ and *η* values of each sample. The pure ZnO nanorods annealed at 500 °C show lower *Q*_uo_ and *Q*_us_ values than the other two pure ZnO nanorods, with *η* decreasing as the annealing temperature increases. However, the Cu(CH_3_COO)_2_-coated ZnO nanorods behave distinctively: all of the three characteristic values increase when the annealing temperature goes up, which implies the photoelectric response and relaxation dynamics of the composite differs from that of the pure ZnO nanorods. It is also notable that *Q*_uo_, *Q*_us_ and *η* are greatly reduced compared to pure ZnO.

**Table 4:** Defined values for all the samples

Defined values	The calculated values for all the samples					
	S400-1	S500-1	S600-1	S400-2	S500-2	S600-2
*Q*_uo_	1.33 × 10^18^	6.58 × 10^17^	1.44 × 10^18^	2.37 × 10^15^	5.19 × 10^15^	2.79 × 10^16^
*Q*_us_	1.17 × 10^18^	5.57 × 10^17^	1.19 × 10^18^	8.64 × 10^14^	2.20 × 10^15^	1.66 × 10^16^
*η* (%)	88.25	84.58	82.56	36.51	42.36	59.40

**Fig. 8:**
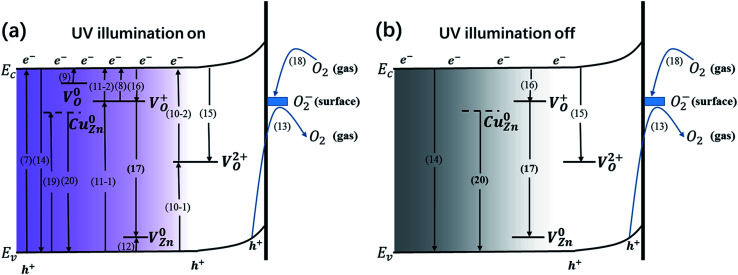
(a) Thorough photogeneration and consumption processes of the photogenerated electrons with UV illumination on, including: electron–hole pair photogeneration *via* a band-to-band transition (7); electron–hole pair recombination *via* a band-to-band transition (14); photoionization of V_O_^+^, sub-process (8); photogenerated electrons are captured by photoionized V_O_^+^, sub-process (16); indirect excitation by V_O_^+^, sub-process (11); photoionization of V^0^_O_, sub-process (9); indirect excitation by V_O_^2+^, sub-process (10); indirect recombination *via* V_O_^2+^, sub-process (15); photoionization of V^0^_Zn_, sub-process (12); oxygen desorption by surface trapped holes, sub-process (13); photogenerated electrons are captured by surface adsorbed oxygen molecules, sub-process (18); donor–acceptor transition involving V_O_^+^ and V_Zn_^−^, sub-process (17); photoexcitation of Cu^0^_Zn_, sub-process (19); the recombination of free holes and electrons captured by Cu^0^_Zn_, sub-process (20). (b) Thorough consumption processes of photogenerated electrons with UV illumination off, including: electron–hole pair recombination *via* a band-to-band transition (14); indirect recombination *via* V_O_^2+^, sub-process (15); photogenerated electrons are captured by photoionized V_O_^+^, sub-process (16); donor–acceptor transition involving V_O_^+^ and V_Zn_^−^, sub-process (17); photogenerated electrons captured by surface adsorbed oxygen molecules, sub-process (18); the recombination of free holes and electrons captured by Cu^0^_Zn_, sub-process (20).

Generally, both the defects and the surface oxygen play a vital role in the generation and recombination of carriers, sequentially impacting the capacity of generating and storing charges during the processes of photoresponse and decay. Thorough photogeneration and consumption processes of the photogenerated electrons with UV illumination on and off are shown in Fig. 8. During the UV illumination, in addition to band-to-band transition (eqn (7)), sufficient carriers can also be produced through donor photoionization (eqn (8) and (9)) and indirect excitation by means of V_O_^2+^ (eqn (10)) and V_O_^+^ (eqn (11)). Besides the above photoexcitation process, the acceptor defect V^0^_Zn_ is similarly excited (eqn (12)). Meanwhile, the desorption of O_2_^−^ takes place (eqn (13)).

7 ZnO + *hv* → e^−^ + h^+^

8 V_O_^+^ + *hv* → V_O_^2+^ + e^−^

9 V^0^_O_ + *hv* → V_O_^+^ + e^−^

10 V_O_^2+^ + e^−^ → V_O_^+^, V_O_^+^ → V_O_^2+^ + e^−^

11 V_O_^+^ + e^−^ → V^0^_O_, V^0^_O_ → V_O_^+^ + e^−^

12 V^0^_Zn_ + *hv* → V_Zn_^−^ + h^+^

13 O_2_^−^ + h → O_2_

When the UV light is off, carriers will disappear through direct recombination (eqn (14)) and indirect recombination assisted by V_O_^2+^ (eqn (15)). The photoionized V_O_^+^ traps the free electrons (eqn (16)). The donor–acceptor transition involving V_O_^+^ and V_Zn_^−^ rapidly consumes carriers (eqn (17)). O_2_ will absorb at the surface (eqn (18)). Since V^0^_O_ has a small capture cross section, the possibility of electrons being captured by V^0^_O_ is slim, signifying that V^0^_O_ might be the centre for charge storage. Obviously, those processes come up during the UV illumination. When it comes to the Cu(CH_3_COO)_2_-coated ZnO nanorods, some extra processes are involved: the photoexcitation of Cu_Zn_ (eqn (19)) and the capture of holes assisted by the defect (eqn (20)).

14 e^−^ + h^+^ → recombination

15 V_O_^2+^ + 2e^−^ → V^0^_O_

16 V_O_^2+^ + e^−^ → V_O_^+^

17 V_O_^+^ + V_Zn_^−^ → V_O_^2+^ + V^0^_Zn_

18 O_2_ + e^−^ → O_2_^−^

19 Cu^0^_Zn_ + *hv* → Cu_Zn_^−^ + h^+^

20 Cu_Zn_^−^ + h → Cu^0^_Zn_

The extent of the impact of the defects and the surface oxygen molecules on the current or the three characteristic values is different. Specifically, surface oxygen acts as an electron acceptor more significantly in the pure ZnO nanorods than in the copper acetate-coated ZnO. As shown in Table 2, the pure ZnO nanorods annealed at 500 °C (S500-1) possess the highest value of surface oxygen (Oe) compared to the other two pure ZnO samples, which accounts for the lowest dark current and the fewest charges during both the photoelectric response and relaxation for S500-1. However, for the copper acetate-coated ZnO nanorods, the samples annealed at different temperatures exhibit similar surface oxygen values (shown in Table 2), indicating the defects and the heterogeneous interface are the main reason for the different photocurrent.

For the samples annealed at the higher temperature, more defects are formed with the enhancement of processes (8)–(11), which are responsible for the higher *Q*_uo_ and *Q*_us_ values. Since the surface oxygen molecules act as electron acceptors, they are able to capture the free electrons from the samples and thus impair the production of carriers. Due to the high density of absorbed oxygen in the pure ZnO nanorods annealed at 500 °C (shown in Table 2), they obtain the least amount of charges during both the photoelectric response and relaxation, which has already been mentioned above. While S400-1 and S600-1 exhibit the same amount of absorbed oxygen, S400-1 has fewer defects than S600-1, which explains the higher *Q*_uo_ and *Q*_us_ values. For the Cu(CH_3_COO)_2_-coated ZnO nanorods, Cu_Zn_ acceptors are likely to compensate for the electrons, leading to smaller *Q*_uo_ and *Q*_us_ values. Since the long term charge storage might stem from V^0^_O_ in the pure ZnO nanorods, *Q*_uo_, *Q*_us_ and *η* will increase with more V^0^_O_. Nevertheless, the recombination of carriers is assisted by V_O_^2+^, V_O_^+^ and *V*_Zn_ (eqn (15)–(17)) when the UV light is turned off, resulting in a negative correlation between the density of the three kinds of defects and *η* in the pure ZnO nanorods: the more defects that exist, the worse the capacity for storing charges is. However, the introduction of Cu_Zn_ acceptors has an impact on the process of carrier recombination: the electrons excited to Cu_Zn_ by UV light are non-conductive and can trap holes upon UV termination, giving fewer opportunities for the recombination of electrons on the CB and holes. As a result, the more Cu_Zn_ acceptors there are, the bigger *η* is in the Cu(CH_3_COO)_2_-coated ZnO nanorods. Furthermore, annealing samples at a high temperature could well favour the production of CuO, greatly influencing the three defined values. It is commonly believed that the interface between two different semiconductors brings out considerable surface states which may trap or diffuse carriers, also accounting for the lower *Q*_uo_ and *Q*_us_ values in the Cu(CH_3_COO)_2_-coated ZnO nanorods in comparison to the pure ZnO nanorods. At the same time, effective separation of the electrons and holes can also be achieved by the space charge region established owing to the gradient of carrier concentration at the interface.^37–41^ Raising the annealing temperature tends to eliminate the surface state as well as promoting the pyrolysis of copper acetate, which makes the space charge region more dominant, leading to more effective charge storage.

## 4 Conclusions

In this report, we provided a whole new perspective on the property of PPC which concerns the storage of photogenerated charges after illumination, possibly affecting optical energy storage in materials showing PPC. More importantly, based on the photocurrent–time curve, a method to represent the charge storage quantitatively was put forward for the first time using the definition of current, with three main values involved: *Q*_uo_, *Q*_us_ and *η*. To validate the method, pure ZnO nanorods and Cu(CH_3_COO)_2_-coated ZnO nanorods, both annealed at different temperatures, were prepared and all of the defined values were calculated from the photocurrent–time curves. Combined with the characterization results of the microstructure, it can be concluded that defects and surface properties have a great influence on the generation and storage of photogenerated charges, both of which can be regulated by synthetic processes and surface modification. V^0^_O_ is considered as the origin of PPC, making it a possible candidate for the centre of charge storage. The absorbed oxygen always impedes the obtainment of charges. V_O_^+^ and V_O_^2+^, whose formations are favored by raising the annealing temperature, are able to provide extra paths for the generation of charges and become effective traps or recombination centres, augmenting *Q*_uo_ and *Q*_us_ but diminishing *η*. Coating the ZnO nanorods in an ethanol solution of copper acetate brought out Cu dopants and even produced CuO as well as sufficient surface states. Though the Cu dopant decreases *Q*_uo_ and *Q*_us_, it has a positive effect on improving *η*. The production of CuO exhibits two opposite effects on *η*: the space charge region between the interface of ZnO and CuO enhances the ability to store charge, while surface states may accelerate the recombination of carriers, reducing *η*. Although evidence of V^0^_O_ being the charge storage centre is somewhat weak, the results still offer us further insight into harnessing PPC to obtain devices with high energy density and capacity to store optical energy for a long time.

## Conflicts of interest

There are no conflicts of interest to declare.

## Supplementary Material

RA-008-C8RA02318B-s001
