# Supplementary material for: Quantitative characterization of the long-term charge storage of a ZnO-based nanorod array film through persistent photoconductance
Source: RSC Adv. 2018 May 4;8(30):16455–63. doi: 10.1039/c8ra02318b (PMC9080277; doi:10.1039/c8ra02318b)
Supplement: RA-008-C8RA02318B-s001 [file RA-008-C8RA02318B-s001.pdf]

Electronic Supplementary Material (ESI) for RSC Advances.
This journal is © The Royal Society of Chemistry 2018

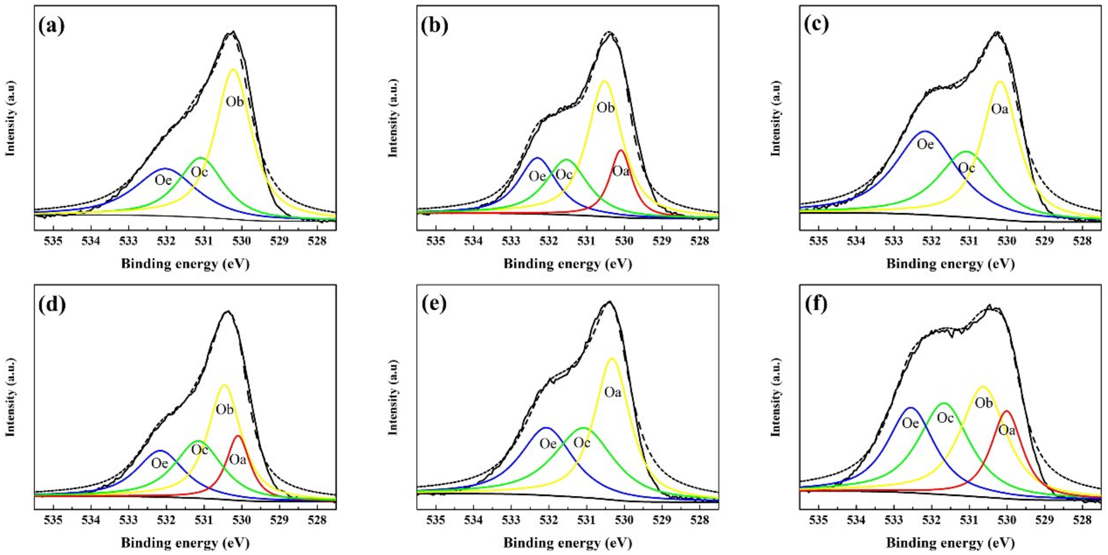

Fig. S1 XPS spectra of O 1s core level of (a)-(c): pure ZnO nanorods annealed at 400, 500, 600°C respectively; (d)-(e): $Cu(CH_3COO)_2$-coated ZnO nanorods annealed at 400, 500, 400°Crespectively.

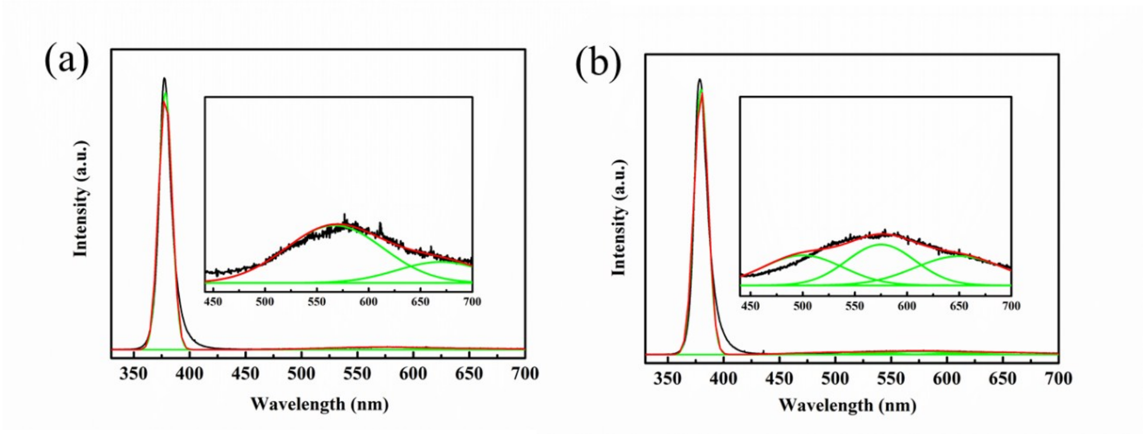

Fig. S2 PL spectra of samples annealed at 400°C: (a) pure ZnO nanorods; (b) $Cu(CH_3CO)_2$-coated ZnO nanorods.

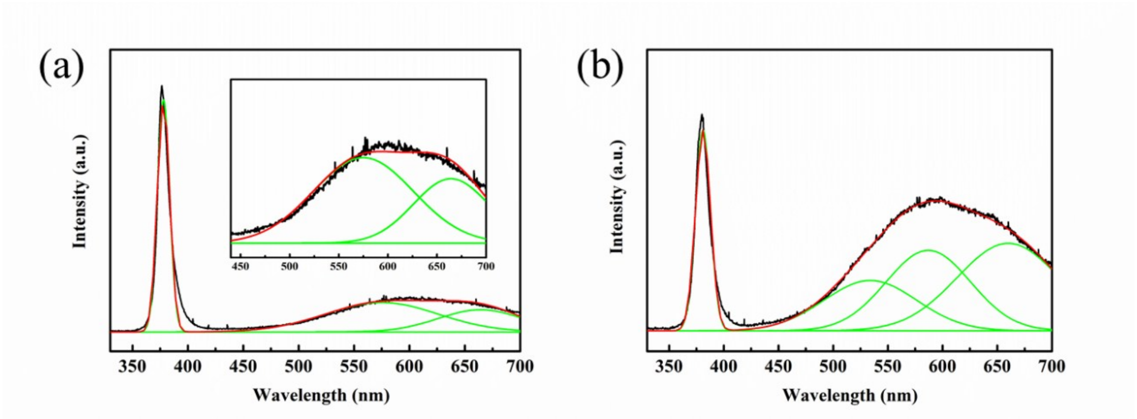

Fig. S3 PL spectra of samples annealed at 500°C: (a) pure ZnO nanorods; (b) $Cu(CH_3CO)_2$-coated ZnO nanorods.

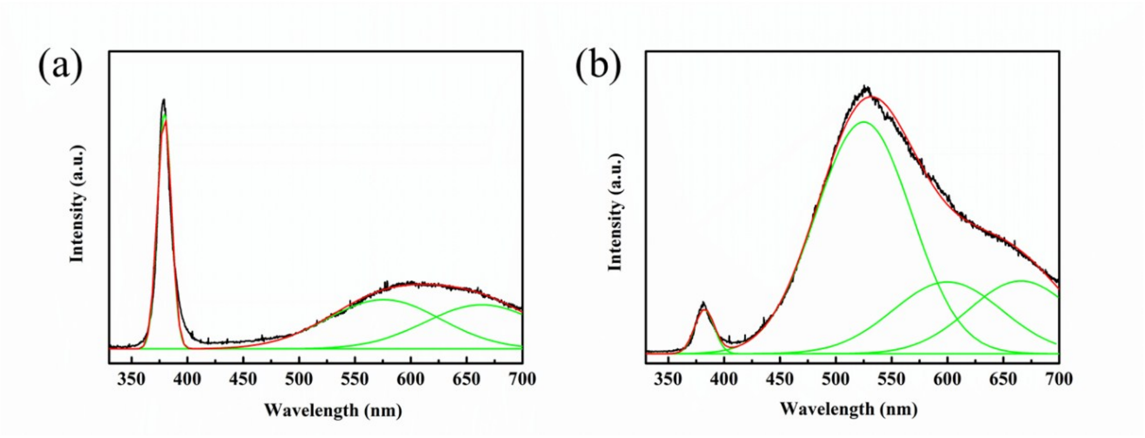

Fig. S4 PL spectra of samples annealed at 600℃: (a) pure ZnO nanorods; (b) $Cu(CH_3CO)_2$-coated ZnO nanorods.
